# Maize haplotype with a *helitron*-amplified cytidine deaminase gene copy

**DOI:** 10.1186/1471-2156-7-52

**Published:** 2006-11-09

**Authors:** Jian-Hong Xu, Joachim Messing

**Affiliations:** 1 Waksman Institute of Microbiology, Rutgers University, Piscataway, NJ 08854, USA

## Abstract

**Background:**

Genetic maps are based on recombination of orthologous gene sequences between different strains of the same species. Therefore, it was unexpected to find extensive non-collinearity of genes between different inbred strains of maize. Interestingly, disruption of gene collinearity can be caused among others by a rolling circle-type copy and paste mechanism facilitated by *Helitrons*. However, understanding the role of this type of gene amplification has been hampered by the lack of finding intact gene sequences within *Helitrons*.

**Results:**

By aligning two haplotypes of the *z1C1 *locus of maize we found a *Helitron *that contains two genes, one encoding a putative cytidine deaminase and one a hypothetical protein with part of a 40S ribosomal protein. The cytidine deaminase gene, called *ZmCDA3*, has been copied from the *ZmCDA1 *gene on maize chromosome 7 about 4.5 million years ago (mya) after maize was formed by whole-genome duplication from two progenitors. Inbred lines contain gene copies of both progenitors, the *ZmCDA1 *and *ZmCDA2 *genes. Both genes diverged when the progenitors of maize split and are derived from the same progenitor as the rice *OsCDA1 *gene. The *ZmCDA1 *and *ZmCDA2 *genes are both transcribed in leaf and seed tissue, but transcripts of the paralogous *ZmCDA3 *gene have not been found yet. Based on their protein structure the maize CDA genes encode a nucleoside deaminase that is found in bacterial systems and is distinct from the mammalian RNA and/or DNA modifying enzymes.

**Conclusion:**

The conservation of a paralogous gene sequence encoding a cytidine deaminase gene over 4.5 million years suggests that *Helitrons *could add functional gene sequences to new chromosomal positions and thereby create new haplotypes. However, the function of such paralogous gene copies cannot be essential because they are not present in all maize strains. However, it is interesting to note that maize hybrids can outperform their inbred parents. Therefore, certain haplotypes may function only in combination with other haplotypes or under specialized environmental conditions.

## Background

Gene sequences of closely related plant species as heterologous markers of genetic maps made it possible to align chromosomal regions of these species according to the order of genes conserved through speciation, also called orthologs [1]. Although early genomic sequence analysis of small orthologous regions of closely related grass species already indicated that gene order is interrupted by genes that have moved around the genome [2-4], the extent of gene mobility during or after speciation could not be appreciated until the comparisons of larger chromosomal intervals from maize and rice [5]. While the extent of non-collinearity among closely related plant species was surprising in view of the comparison of closely related mammalian species, it was always assumed that within the same species gene order was preserved. The conserved gene order within the same species has been the mainstay of genetic maps because meiotic recombination occurs primarily within genes [6,7] or unique, but complete homologous sequences [8].

However, several comparative sequence analyses among maize inbred lines have now shown that haplotype variability within the same species did not only differ by transposable element insertions but also by the presence of non-allelic gene copies [9-11]. Although retrotranspositions account for major dissimilarities between aligned regions, more than one-third of the predicted genes (27/72) are absent in one of the inbreds (B73 and Mo17) at the loci 9002, 9008 and 9009 [11]. However, in all these cases the inserted genes are incomplete and represent gene fragments except at the *z1C1 *locus in BSSS53 [10]. At the *z1C1 *locus, B73 has lost four gene copies of the zein gene family that are present in BSSS53, while BSSS53 has gained three additional copies compared to B73. One of the lost genes and two of the added genes are functional and expressed. Moreover, the two new genes have changed in their transcriptional regulation [12]. While the change in copy number of tandemly arranged genes is not unexpected, there were also insertions of unlinked genes. B73 had the insertion of a RAV-like B3-domain DNA-binding protein putative gene, conserved on rice chromosome 3 (1e-143) and BSSS53 has the insertion of two genes, a putative cytidine deaminase gene and a hypothetical gene encoding part of a 40S ribosomal protein [10]. While the RAV-like gene in B73 is a gene fragment linked to a transposable element (Xu and Messing, unpublished data), reminiscent of the Pack-MULEs [13], the two genes in BSSS53 appear to have complete open reading frames.

At this point, it is unclear how widespread non-collinearity is among cultivars of other species. Comparison of orthologous regions of two subspecies of rice, *O. sativa *japonica and indica, in one study did not show insertion or deletions of genes or gene fragments [14]. However, comparison of rice chromosome 4 in those two subspecies shows examples of insertion and deletion of genes [15]. Comparison of orthologous regions around the *Rph7 *locus of two barley cultivars also differed by at least one gene that could encode a helicase [16]. Therefore, it appears that maize inbred lines stand out in the degree of intra-species gene non-collinearity among other species studied so far. The underlying question is what is the selective advantage of gene mobility and what is the mechanism that allows genes to move from one location to another?

One could hypothesize that moving genes into a different chromosomal context could provide novel regulation of an existing gene function or produce novel gene function [17]. If it results from a "copy and paste" rather than a "cut and paste" mechanism, it also could provide redundancy of a critical function or could contribute to one of many quantitative trait loci (QTLs). Recently, it was shown that the imprinted gene *Peg10*, important for early mouse development, is inserted into a retrotransposon [18]. Similarly, a gene expressed during seed development [19], the maize *Fie2 *gene, is also inserted into a retrotransposon [20]. Since in both cases the genes contain introns and are single copy genes, they could not have moved there by retrotransposition. Both of these cases are likely derived from a "cut and paste" mechanism, which is different to the examples of non-collinear genes described in this study. Sequence alignments of allelic and non-allelic gene copies have shown that these insertions except for the *z1C1 *genes were due to a novel type of DNA transposition that is based on a "copy and paste" mechanism [21,22]. Because this mechanism requires a helicase to initiate a rolling circle-type of replication, the transposed unit is referred to as *Helitron*.

The *Helitrons *were first identified by computational analysis in *Caenorhabditis elegans*, *Arabidopsis thaliana *and *Oryza sativa *[23]. *Helitrons *have conservative 5'-TC and CTRR-3' and do not have terminal inverted repeats and target site duplications. Instead, they contain 16- to 20-bp hairpins 10–12 nucleotides upstream from the 3'-end and transpose precisely between the 5'-A and 3'-T, with no modifications of the AT target sites. Autonomous *Helitrons *encode a 5'-to-3' DNA helicase and nuclease/ligase similar to those encoded by known rolling-circle replicons. Together with their multiple diverged non-autonomous descendants, *Helitrons *constitute 2% of both the *Caenorhabditis elegans *and *Arabidopsis thaliana *genomes. In maize, non-autonomous *Helitron *insertions carrying gene fragments are responsible for loss-of-function mutations in the *shrunken2 *gene and the *barren stalk1 *gene [24,25]. Examination of donor and target sites between two inbred lines of maize have then illustrated that at the donor site a portion of an intact gene is copied and inserted into an unlinked genomic location. While the donor site is conserved with allelic versions of a regular gene in different maize inbreds, the target site creates a disruption in gene order and a non-allelic gene copy [22]. In all cases so far the transposed genes by *Helitrons *are gene fragments or pseudogenes [21].

Here, we report an example where a *Helitron *contains an intact gene that encodes a putative cytidine deaminase (CDA). Cytidine deaminases are a superfamily, which includes Cytidine deaminase, nucleoside deaminase, deoxycytidylate deaminase and riboflavin deaminase. They are enzymes that de-aminate cytidine to uridine and play an important role in a variety of pathways from bacteria to man. Ancestral members of this superfamily were only able to de-aminate cytidine of mononucleotides or nucleosides. Recently, a family of enzymes, the APOBEC family of mRNA editing enzymes, has been discovered that have the ability to de-aminate cytidines not only of RNA [26,27], but also of DNA [28,29]. However, the role of CDAs in plants is still poorly understood. A CDA-encoding gene from Arabidopsis has been characterized as a housekeeping gene [30], but whether there are CDA genes with other functions and in other plant species still needs to be explored. We, therefore, took advantage of the variability of maize haplotypes to examine not only the contribution of *Helitron *action to CDAs in maize inbred lines but also the organization of CDA-encoding genes in the maize genome, their expression, and their putative function by their phylogenetic relationship to other CDAs.

## Results and Discussion

### A new *Helitron *at the *z1C1*+*BSSS53 *locus

To unravel the mechanisms by which haplotypes of maize acquire the insertion of new sequences, we took advantage of the recently sequenced regions of the *z1C1 *locus on chromosome 4 from two inbred lines. The chromosomal region from BSSS53 is a contiguous sequence of 435,076 bp [GenBank:AF528565, AF090447] and from B73 263,630 bp [GenBank:AC144717, AC144718]. When these two sequences are aligned, the two intervals differ by about 100 kb in size. Expansion of the BSSS53 region is in part due to additional retrotransposition events and in part due to gene insertions [10]. Insertions can be classified by their target site sequences. We therefore aligned the B73 sequences surrounding the insertion in the BSSS53 sequence to determine the end points of the insertion and the sequences at the ends of the insertions.

**Figure 1:**
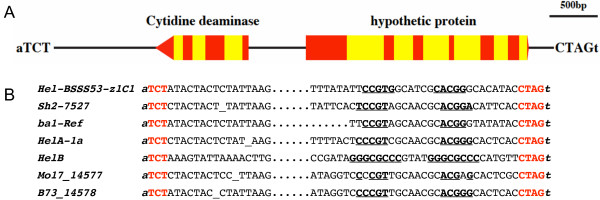
***Hel-BSSS53-z1C1 *on maize chromosme 4S**. A) A physical map of *Hel-BSSS53-z1C1 *is presented. Putative genes are shown as pentagons pointing in the direction of transcription. Exons are shown in red and introns in yellow. Conserved nucleotides at the 5'-TCT and 3'-CTAG termini and 5'-a, 3'-t target sites are highlighted at the ends. B) Termini of the maize *Helitrons Hel-BSSS53-z1C1 *from BSSS53 4S [10], *HelA-1a *and *HelB *from McC 9S [22], *Mo17_14577 *from Mo17 9L, *B73_14578 *from B73 6S [11], the *Helitron *insertions in mutants *sh2-7527 *[24] and *ba1-Ref *[25, 49]. *Helitron *sequences are in uppercase letters and the invariant host nucleotides, where the *Helitrons *inserted, are in *italic *lowercase letters. Conserved nucleotides at the 5' and 3' termini are in red bold uppercase letters and inverted repeats at the 3' termini are underlined.

Transposable elements were easily recognized by their target site duplications, but the amplification of zein genes in BSSS53 did not show any specific target site sequences. However, the result was different for the presence and absence of putative genes that could encode a cytidine deaminase and a partial 40S-ribosomal protein. This insertion in BSSS53 is 5,233 bp long and exhibits the hallmarks of a *Helitron *[23,24]. Based on sequence alignment of the two inbreds the insertion occurred between the AT target sequence. It lacks the target site duplication seen for transposable elements, but it has the typical 5'-TCT and 3'-CTAG ends. However, coding information for the helicase is lacking and replaced by one putative intact gene encoding a cytidine deaminase (CDA) and a hypothetical gene encoding a part of an unknown and part of a 40S-ribosomal protein (Fig. 1A).

To further characterize this insertion in BSSS53, we aligned the end sequences with the end sequences of other known *Helitrons *in maize. In addition to the 5' and 3' end sequences we also can find a 16-bp region capable of forming a hairpin structure that is conserved among the maize *Helitrons *(Fig. 1B). We named this insertion *Hel-BSSS53-z1C1 *(*Hel*itron in maize *BSSS53z1C1*region). While the sequence features of the end sequences of the BSSS53 insertion are conserved with the other *Helitrons*, it differs from them by having putative genes with complete coding potential.

### Organization of cytidine deaminase (CDA) genes in maize inbred lines

**Figure 2:**
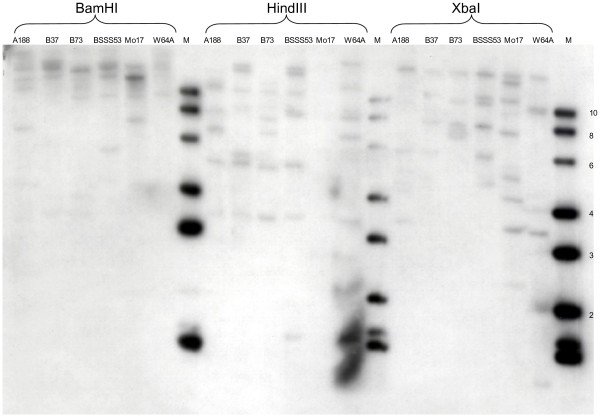
**Analysis of genomic CDA-related sequences in maize inbred lines**. Genomic DNA from various inbred lines have been digested with *BamHI*, *HindIII *and *XbaI *and subjected to Southern blot analysis as described under Methods. The names of each inbred line are marked above the lanes.

Because the hallmark of *Helitrons *is a "copy and paste" mechanism, one would expect that both B73 and BSSS53 would share a common donor site for the sequences contained in *Hel-BSSS53-z1C1*. We decided to select the CDA gene sequence rather than the sequence for the hypothetical gene encoding part of the 40S ribosomal protein for such an analysis because of the value of an intact gene with a known function for tracing *Helitron *action. We first cloned a CDA cDNA clone based on the CDA gene sequences in the *Helitron *by reverse transcription of leaf mRNA (Methods) and used it in Southern blot analysis of these two inbreds along with other common inbreds A188, B37, Mo17, and W64A (Fig. 2). As restriction enzymes are chosen that do not cleave within the maize CDA genomic sequence, we can find evidence for a small multigene family as it has been observed in Arabidopsis as well [30]. Therefore, screening of a putative donor site of the CDA gene of the *Helitron *required a high stringency to filter out CDA genes that are distantly related and possibly have different specificities.

Higher stringency was afforded by BLAST search of BAC end sequences (BESs) with the CDA gene of the *Helitron *[31]. BESs are anchored to the genetic map through the fingerprinted B73 physical map [32,33]. A single BAC contig #299 on chromosome 7 was identified and a clone, b0390I10, was selected for sequencing that contained the CDA sequence in the center of the clone. Indeed, b0390I10 contained a putative gene encoding a complete CDA [GenBank: EF106973]. If this putative gene on chromosome 7 is the original maize CDA donor gene, one would expect it to be present in all inbred lines. We therefore constructed a library of genomic sequences from 15 common inbreds that have been amplified with a set of primers of CDA sequences conserved between the chromosome 7 and 4 locations. From each inbred 24 clones were sequenced to quantify the number of genes present in each genome by sequence cluster analysis and insert size [34]. Based on this analysis we obtain four different CDA gene copies, which represent a subset of those detected by Southern blot analysis. Only two genes were present in all inbred lines (Table 1) including the one of clone b0390I10 on maize chromosome 7. We named this CDA gene *ZmCDA1*. It is not unexpected to find two copies in all inbred lines because maize formed by the hybridization of two closely related progenitors 4.8 mya [37]. We therefore named the second gene copy that is present in all inbred lines *ZmCDA2*. While B73 seems to have only these two copies of the CDA gene, other inbred lines like BSSS53, Mo17, A654, B37 and CO159 have an additional copy that appears to be in common. Based on sequence cluster analysis this copy corresponds to the chromosome 4 location of BSSS53. We named this copy *ZmCDA3*, which is the one contained in the *Hel-BSSS53-z1C1 Helitron*. In addition, we can find at least one more copy that is also present in A188, BSSS53, Tx303, CO159, CM37, W64A, Mo17, A632 and A636. However, this copy appears to have a 39-bp deletion in the last exon. Additional truncated copies could be present in some of the inbreds as well. Therefore, the only intact genes of this subfamily of CDA genes in maize are *ZmCDA1*, *ZmCDA2*, and *ZmCDA3*.

**Table 1:** Summary of *ZmCDA *patterns in 15 maize inbred lines*.

	A632	A636	A654	B37	Mo17	W22	W23	W64A	CM37	R232	CO159	Tx303	A188	B73	BSSS53
*ZmCDA1*	+	+	+	+	+	+	+	+	+	+	+	+	+	+	+
*ZmCDA2*	+	+	+	+	+	+	+	+	+	+	+	+	+	+	+
*ZmCDA3*	-	-	+	+	+	-	-	-	-	-	+	-	-	-	+
Truncated	+	+	-	-	+	-	-	+	+	-	+	+	+	-	+

* Sequences of amplified genomic DNA from each inbred with primer pairs ZmCDAF3/ZmCDAR3 clustered as consensus sequences; presence of consensus sequence (+), absence of consensus sequence (-).

### Relationship of CDA genes in maize

**Figure 3:**
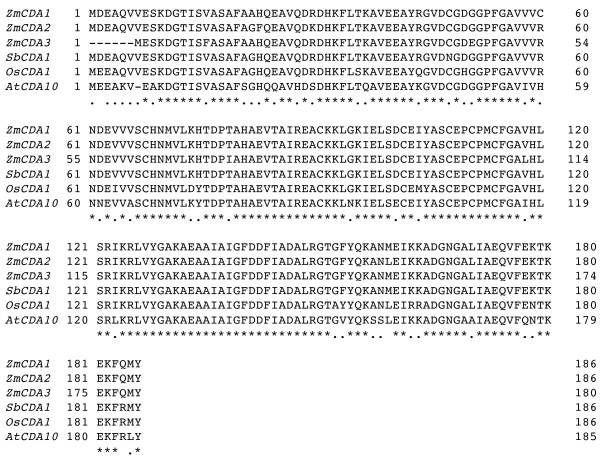
**Alignment of amino acid sequences of related CDAs from maize, sorghum, rice, and Arabidopsis**. The coding sequences of CDAs from maize and other organisms have been translated into single letter amino acid code and aligned using the Laser gene program as described under Methods. Gene names are marked at the beginning of the sequence. Conserved amino acid positions are highlighted by stars (*).

**Figure 4:**
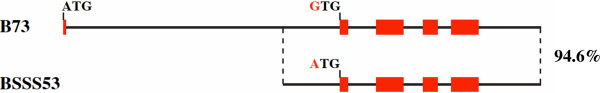
**Alignment of the *ZmCDA1 *and *ZmCDA3 *genes**. The coding sequences of the *ZmCDA1 *and *ZmCDA3 *genes from B73 and BSSS53 are depicted graphically as bars to illustrate their common sequences. The start codons are highlighted. Exons are shown in red block, nucleotide change from G to A is shown in red letters.

We used the *ZmCDA1 *sequence for a BLAST search [31] of the rice genome, Arabidopsis genome, and the sorghum EST collections. We could identify one copy [GenBank: AAR88587] on rice chromosome 3, which we named *OsCDA1*. We also identified one related sequence on Arabidopsis chromosome 5, which we named *AtCDA10*, and one related sequence from sorghum EST data [GenBank: CN133157.1, gi:45963787], which we named *SbCDA1*. The three maize genes and the related sequences from rice, Arabidopsis, and sorghum were translated and their amino acid sequences compared (Fig. 3). Because rice chromosome 3 contains orthologous regions to maize chromosome 7 [35], it is likely that the rice *OsCDA1 *gene and the maize *ZmCDA1 *gene are orthologous and derived from a common ancestral chromosome. If that is the case, then *ZmCDA1 *and *ZmCDA2 *should be closer to rice than *ZmCDA3*. Indeed, based on amino acid homology *ZmCDA1 *and *ZmCDA2 *are closer to rice than *ZmCDA3 *(not shown). Because *ZmCDA3 *appears to be closer to *ZmCDA1 *than to *ZmCDA2*, the *Helitron *has probably copied *ZmCDA1 *rather than *ZmCDA2*. To further analyze the relationship of *ZmCDA1 *and *ZmCDA3*, their genomic sequences were aligned as well. From this comparison, it appears that the deletion of the amino-terminal amino acids is due to a deletion of a small part of the first exon and a larger part of the first intronic sequence of *ZmCDA1 *(Fig. 4). Interestingly, a putative de-amination of the seventh codon restored an ATG start codon and a complete open reading frame for the *ZmCDA3 *gene. Otherwise, the aligned sequences including the introns are highly conserved (94.6%). From this analysis, it appears that the progenitors of rice and maize had a common CDA gene that got duplicated during the speciation of maize. The two copies are therefore orthologous gene copies. Subsequently, a *Helitron *initiated the replication of one of the copies, the one on maize chromosome 7. During this process the 5' region got truncated and a new start-codon generated. This third copy got integrated into a new chromosomal location on maize chromosome 4 along with other sequences including those that are part of a 40S ribosomal protein gene. The third CDA gene copy is paralogous and arose after speciation and therefore contributes to a unique haplotype.

### Distance analysis of CDA genes

If the chronology of events is correct, we can determine the timing of these events based on the nucleotide substitutions of each gene pair. As molecular studies have suggested an upper bound of 50 million years ago (mya) for the origin of rice [36], we make the assumption that the progenitors of maize and sorghum separated from the progenitor of rice 50 mya. Using this divergence date, we estimated the average nucleotide substitution rate for the maize CDA genes and determined the distance between the *ZmCDA1 *and *ZmCDA2 *genes. They appear to have diverged about 14.5 mya, which is higher than the 11.9 mya observed previously [37]. However, the previous study also found that synonymous distances between different orthologs of maize and sorghum could vary 3.2-fold. If we calculate the distance of maize and rice CDA orthologs, they would have diverged 12.8 mya. Therefore, the maize CDA orthologs are within the same range of nucleotide substitution rates as previously studied ones and support the allotetraploid model of the origin of the maize genome.

**Figure 5:**
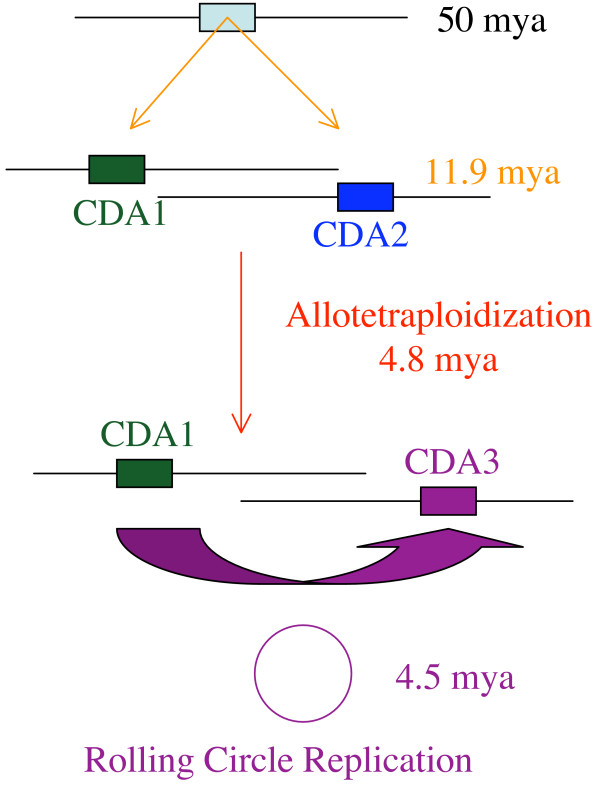
**Origin of the *ZmCDA1*, *ZmCDA2*, and *ZmCDA3 *genes**. The progenitors of rice and maize split about 50 mya and the two progenitors of maize about 11.9 mya. Each of the progenitors of maize contain a copy of the CDA gene shared with the progenitor of rice (orthologs). When the two progenitors of maize hybridized at about 4.8 mya (allotetraplodization), the maize genome maintained two diverged orthologous CDA gene copies, *ZmCDA1 *and *ZmCDA2*. In addition, 4.5 mya the *ZmCDA1 *copy on chromosome 7 got copied by the action of a helicase and inserted into chromosome 4S close to the *z1C1 *locus, resulting in the *ZmCDA3 *gene copy, which is therefore a paralogous gene copy.

Because the *ZmCDA3 *gene also has an intact coding sequence it provides us for the first time the opportunity to determine when *Helitron *movement occurred. We make the assumption that conservation of an intact gene has the same substitution rate regardless whether is expressed constitutively or under specialized conditions. Based on the synonymous nucleotide substitutions of the *ZmCDA3 *gene relative to the donor gene *ZmCDA1 Helitron *movement occurred about 4.5 mya (Fig. 5), which is just after the hybridization of the progenitors of maize, estimated to be 4.8 mya [37]. Interestingly, it has been shown that the majority of retrotranspositions also occurred after the hybridization of the two progenitors in maize as early as 4.6 mya but with 74% less than one mya [20]. Similarly, paralogous copies of zein genes arose 4.3, 2, and 0.5 mya [12]. Therefore, within the same time frame different mechanisms have triggered amplification and integration of sequences into the maize chromosomes including *Helitrons*.

### Cytidine deaminase gene expression in maize inbred lines

**Figure 6:**
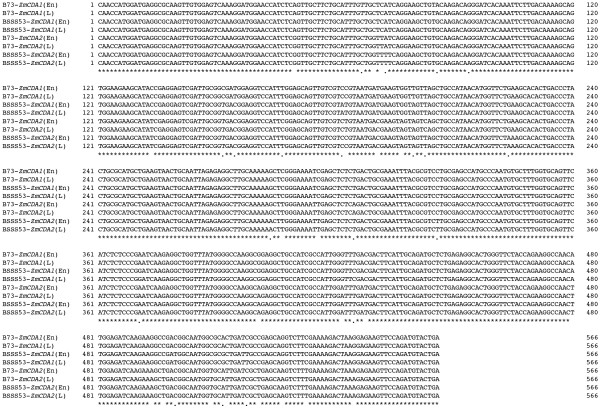
**Alignment of CDA-related maize cDNAs sequences**. Leaf and endosperm tissue samples were used to extract mRNA, which were used to clone CDA-related sequences. They were sequenced and aligned with the genomic coding sequences to determine from which genes they were derived. Alignment was performed using the Laser gene program as described under Methods. Sequences are marked at their beginning.

To investigate whether this subfamily of CDA genes is expressed in maize, we tested tissues for these CDA transcripts. We sampled total mRNA from leaf tissues of adult plants from six typical inbred lines A188, B37, B73, BSSS53, W64A and Mo17 and from 20-days old immature endosperm of BSSS53 and B73. The mRNA was amplified by RT-PCR, cloned and analyzed by sequence analysis [GenBank: EF105328–EF105335]. Alignment of RT-PCR products shows that the mRNA is derived from the *ZmCDA1 *and the *ZmCDA2 *genes (Fig. 6). Having the same genes expressed in all inbred lines is consistent with our genomic analysis of CDA genes of maize inbred lines that have the *ZmCDA1 *and *ZmCDA2 *gene copy in common (Table 1). The expression analysis also indicates that plants have a CDA gene that is conserved through the monocot-dicot divergence and that the Arabidopsis, rice, and maize gene probably originated from the same ancestral gene. However, we could not find a transcript that matches *ZmCDA3*. Although it has a complete open reading frame, it is not unexpected that its regulation has changed because of the new position in the genome. One certainly would predict that its expression could not be vital for plant development because its copy is absent in B73. Therefore, it is quite possible that *ZmCDA3 *is expressed either in very specialized tissue or under specific environmental conditions. Given that this copy arose about 4.5 mya, one would expect that it has maintained its expression potential against deterioration of its sequence. One reason could be that unique haplotypes in the maize genome containing non-allelic gene copies are potentially required for its heterotic properties [10].

### Putative functions of CDAs in maize inbred lines

**Figure 7:**
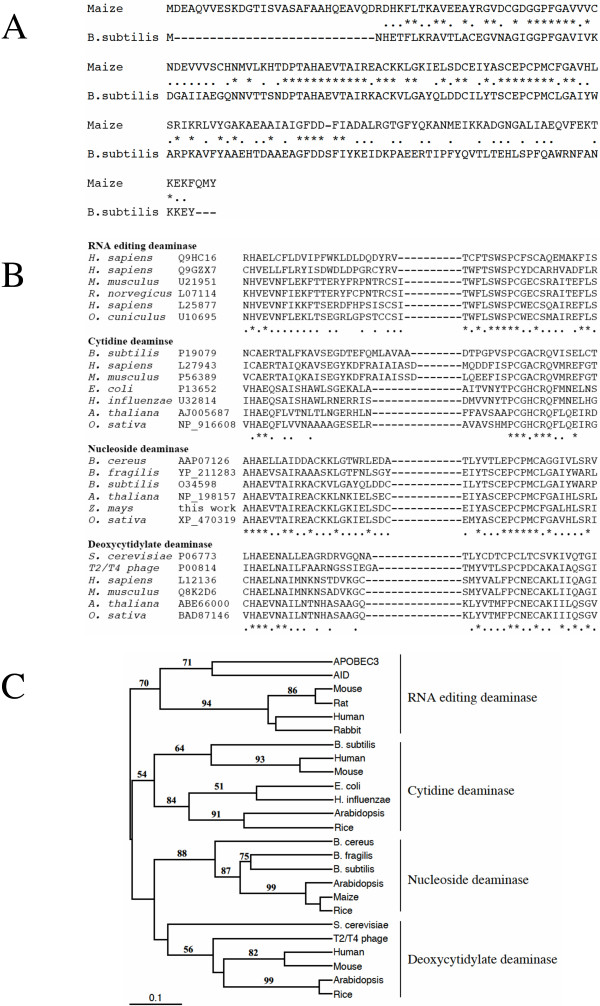
**CDA-related genes according to their function**. A) Alignment of amino acid sequences of maize cytidine deaminase and *B. subtilis* nucleoside deaminase. Identical and conserved amino acids are marked by asterisks and dots, respectively; hyphens represent gaps. B) Alignment of the active sites of cytidine deaminases. Accession numbers of amino acid sequences used here are as follows: [GenBank: APOBEC3, Q9HC16; AID, Q9GZX7]; mouse [GenBank: APOBEC1, U21951]; rat [GenBank: APOBEC1, L07114]; human [GenBank: APOBEC1, L25877]; rabbit [GenBank: APOBEC1, U10695]; *B. subtilis *cytidine deaminase [GenBank: P19079]; human cytidine deaminase [GenBank: L27943]; mouse cytidine deaminase [GenBank: P56389]; *E. coli *cytidine deaminase [GenBank: P13652]; *H. influenzae *cytidine deaminase [GenBank: U32814]; Arabidopsis cytidine deaminase [GenBank: AJ005687]; rice cytidine deaminase [GenBank: NP_916608]; *B. cereus *nucleoside deaminase [GenBank: NP_829925]; *B*. *cereus *nucleoside deaminase [GenBank: AAP07126]; *B. fragilis *nucleoside deaminase [GenBank: BAD48383]; *B. subtilis *nucleoside deaminase [GenBank: O34598]; Arabidopsis nucleoside deaminase [GenBank: NP_198157]; maize nucleoside deaminase, this work [GenBank: EF106973]; rice nucleoside deaminase [GenBank: XP_470319]; *S. cerevisiae *deoxycytidylate deaminase [GenBank: P06773]; T2/T4 phage deoxycytidylate deaminase [GenBank: P00814]; human deoxycytidylate deaminase [GenBank: L12136]; mouse deoxycytidylate deaminase [GenBank: Q8K2D6]; Arabidopsis deoxycytidylate deaminase [GenBank: ABE66000]; rice deoxycytidylate deaminase [GenBank: BAD87146]. C) The phylogenetic tree was constructed based on the active site of cytidine deaminases using the PAUP 4.0b10 UPGMA method [50]. Numbers indicate bootstrap values (above 50% with 1000 replications).

Function of CDAs is quite diverse, but can be classified by protein structure. The cDNAs of *ZmCDA1* and *ZmCDA2* described here encode a protein of 184 residues; *ZmCDA3* would be 6 residues shorter. Homology search against databases of known protein sequences revealed significant homology (39.4% amino acid identity and 74.8% similarity) with *B. subtilis *nucleoside deaminase (Fig. 7A). A database search revealed the presence of an active site for nucleoside deaminases, which is conserved within the cytidine deaminase super-family. The cytidine deaminase super-family can be classified into RNA-editing deaminases, cytidine deaminases, nucleoside deaminases and deoxycytidylate deaminases, based on substrate specificity and homology of the active-site sequence [26]. To characterize the putative function of the CDAs in maize, we performed a phylogenetic analysis of CDAs from different organisms with the active site protein sequences (Fig. 7B). The cladogram shows that the active site of maize genes, the rice and the Arabidopsis genes, form a clade, which is close to the bacterial genes encoding nucleotide-modifying enzymes (bootstrap value of 88), and constitute the nucleoside deaminase family that is distinct from the mammalian genes encoding RNA or DNA modifying enzymes, cytidine deaminase, and deoxycytidylate deaminase families (Fig. 7C). In general, nucleoside deaminases include adenosine, guanine and cytosine deaminases, which catalyze the de-amination of nucleosides. The functional enzyme is a homodimer. Cytosine deaminase catalyzes the de-amination of cytosine to uracil and ammonia. Because it is found in bacteria and fungi and not in mammals the enzyme is currently of interest for antimicrobial drug design and gene therapy applications against tumours [38-41]. Adenosine deaminases generate inosine at the first position of the anticodon (position 34) of specific tRNAs, which is thought to enlarge the codon recognition capacity during protein synthesis. Guanine deaminases de-aminate guanine to xanthine as part of the utilization of guanine as a nitrogen source. Based on the clustering of ESTs from Arabidopsis, another CDA gene, *AtCDA1*, has previously been isolated and its function tested *in vitro *[30]. The *AtCDA1 *gene de-aminates cytidine, but cannot use RNA as a substrate. Therefore, it resembles the enzyme found in *E. coli *with the same function and differs in its structure from the mammalian proteins. Nevertheless, we know that RNA editing occurs in plant organelles [42]. Moreover, biochemical assays have shown that extracts contain an enzyme activity consistent with the RNA-editing function [43]. Because most proteins in chloroplast are derived from the nuclear genome, one would also expect CDA encoding genes in the plant genome that are closer to the mammalian clade of CDAs capable of modifying RNA or even DNA. As the Southern blot of genomic DNA from different inbred lines indicates the maize genome contains other CDA-related gene sequences that could account for these other functions (Fig. 2).

## Conclusion

A new *Helitron *has been discovered whose internal sequences contain an intact coding sequence for a cytidine deaminase. Because previous *Helitron *sequences were missing intact coding sequences it was not possible to determine when they were copied from their donor site. From sequence alignments with the two orthologous copies of the maize genome and the copy of the rice genome, it appears that the *Helitron*-based movement of the paralogous CDA gene copy occurred about 4.5 mya. Conservation of the coding regions including intronic sequences of 94.6% between the orthologous and the paralogous gene copies suggests that the paralogous gene copy is required although not as an essential gene. Therefore, it is likely to be conditionally induced and/or provides a quantitative trait as a selective advantage rather than a typical housekeeping function. One can envision that it is expressed under certain environmental conditions. Another possibility is that it is under the control of a regulatory factor that itself is encoded by a paralogous gene sequence and is only present in unique haplotypes. In such a scenario, one would have to combine these two haplotypes by crossing to enable the expression of the paralogous CDA gene copy. While combinatorial regulatory circuits for a CDA function still remains to be explored, it can serve as a paradigm of how one could achieve unique phenotypes that require the right inbred strains to form a hybrid.

## Methods

### Plant materials

Our laboratory stocks of maize inbred lines A632, A636, A654, B37, Mo17, W22, W23, W64A, CM37, T232, CO159, Tx303, A188, B73, and BSSS53 were obtained from the NPG collection [44].

### Identification of B73 clone b0390I10

The CDA gene sequence linked to the *z1C1 *locus on chromosome 4S of BSSS53 was subjected to a BLAST search [31] of the B73 BESs on our local server. One BAC end sequence [GenBank: CG435284] had homology to the coding region of a CDA gene. We searched the B73 maize physical map for a BAC contig [45] containing the positive clone. Contig #299 on chromosome 7 was identified. However, after positioning the CDA containing BES within the FPC BAC clone b0390I10 was selected for sequencing to obtain all CDA flanking sequences [GenBank: EF106973].

### Southern blot hybridiztion

A total of 25 μg of maize genomic DNA was digested with different restriction enzymes. DNA fragments were separated on 1.0% agarose gels, blotted on Hybond-XL nylon membranes (Amersham Biosciences), and the membranes were hybridized with CDA cDNA sequences as a probe that had been labeled as described previously [46].

### Genomic PCR and Reverse-transcription PCR (RT-PCR)

CDA gene sequences were amplified from total genomic DNA of 15 inbred lines by using primer pairs, covering exon 2, intron 2, exon 3, intron 3, and exon 4:

ZmCDAF3, CATCTGGAACTTCTCCTTAG,

ZmCDAR3, ACAGGGATCACAAATTCTTG;

the last exon, exon 4, of the CDA gene was also amplified with primer pairs:

ZmCDAF4, GACTTCATTGCAGATGCTCTG,

ZmCDAR1, TCAGTACATCTGGAACTTCTC.

Total RNA of mature leaves and immature endosperm 20 days after pollination of maize inbred lines was extracted with the RNeasy Plant Mini Kit (Qiagen). We reverse-transcribed RNA to cDNA using SuperScript First-Strand Synthesis System (Invitrogen) with an oligo(dT) primer.

Amplification was then carried out for the *ZmCDA1 *and *ZmCDA2 *genes with primer pairs:

ZmCDAF1, CAACCATGGATGAGGCGCAAG,

ZmCDAR1, TCAGTACATCTGGAACTTCTC, and for the *ZmCDA3 *gene with primer pairs:

ZmCDAF2, ATGGAGTCAAAGGATGGAAC,

ZmCDAR1, TCAGTACATCTGGAACTTCTC.

### DNA and cDNA sequencing and analysis

The BAC clone was sequenced by DNA shotgun sequencing with an Applied Biosystems 3730 × 1 DNA Analyzer using universal primers [47]. PCR and RT-PCR products were cloned and also sequenced with universal primers [GenBank: EF105328–EF105335]. DNA sequences were analyzed with the Laser gene application kit (DNAstar, Madison, WI). Multiple sequence alignments were carried out by the program ClustalW [34].

### Estimation of the rate of synonymous substitution and divergence time

We used CDA gene exon sequences to estimate synonymous substitution rates (k) using standard methods [48]. The divergence time T between maize, sorghum, and rice was set at 50 million years [36]. The substitution rate k was calculated according to the formula k = [Ks(a)+Ks(b)]/2'2T, where Ks(a) is the relative synonymous substitution rate between maize and rice, and Ks(b) the relative synonymous substitution rate between sorghum and rice.

For estimation of the divergence time t of two sequences, we used t = ds/2k.

## Authors' contributions

J-H. X. performed the experiments and J. M. helped with the design of the experiments and the writing of the manuscript. The final version of the manuscript has been read and approved by all authors.
